# Modeling Inherited Methylmalonic Acidemia Using Isogenic Human Induced Pluripotent Stem Cell-Derived Hepatocytes with Mutations in *Methylmalonyl-CoA Mutase*

**DOI:** 10.17912/micropub.biology.002044

**Published:** 2026-06-17

**Authors:** Behshad Pournasr, Stephen Duncan

**Affiliations:** 1 Regenerative Medicine and Cell Biology, Medical University of South Carolina, Charleston, SC, United States; 2 Department of Biology,, Citadel, Charleston, SC, United States; 3 Regenerative Medicine and Cell Biology, Medical University of South Carolina, Charleston, SC, US

## Abstract

Isolated methylmalonic acidemia (MMA) is an autosomal recessive disorder that increases methylmalonic acid by affecting the metabolism of propionyl-CoA. Mutations in multiple genes cause MMA, however, those in methylmalonyl-CoA mutase ( *MMUT* ) are most common. MMUT is an isomerase that converts methylmalonyl-CoA to succinyl-CoA, a key tricarboxylic acid (TCA) cycle intermediate. The defects in these metabolic processes disproportionately affect hepatocytes in patients with MMA. We performed gene editing in human induced pluripotent stem cells (hiPSCs) to generate loss-of-function mutations within *MMUT* . Hepatocytes derived from these iPSCs recapitulate key aspects of methylmalonic acidemia, providing a new model of MMA.

**Figure 1. Generation and Characterization of MMUT–deficient iPSCs:** (A) Schematic illustration of *MMUT* gene structure highlighting exon 3, sequence of CRISPR/Cas9 guide (red arrow), position of the PAM element, and the nucleotide sequences of wild-type ( *MMUT* ^+/+^ ) and mutant alleles ( *MMUT* ^EX3Δ17/+2^ , *MMUT* ^EX3Δ10/+1^ , and *MMUT* ^EX3Δ31/+1^ ). (B) Image of a polyacrylamide gel displaying *MMUT* exon 3 amplicons from wild-type control ( *MMUT* ^+/+^ ) iPSCs and those harboring mutations ( *MMUT* ^EX3Δ17/+2^ , *MMUT* ^EX3Δ10/+1^ , and *MMUT* ^EX3Δ31/+1^ ). (C) Fluorescent immunostaining of day 20 hepatocytes derived from wild type ( *MMUT* ^+/+^ ) and mutant iPSC lines ( *MMUT* ^Ex3Δ17/+2^ , *MMUT* ^Ex3Δ10/+1^ , *MMUT* ^Ex3Δ31/+1^ ) for Albumin (Red), HNF4a (green) and Methylmalonyl-CoA Mutase (MUT, Green), scale bars = 100 µM (Albumin and HNF4a) and 80µM (MUT). (D) Western blot analysis to detect Methylmalonyl-CoA Mutase protein (MUT) in either wild-type ( *MMUT* ^+/+^ ) or mutant ( *MMUT* ^EX3Δ17/+2^ , *MMUT* ^EX3Δ10/+1^ , and *MMUT* ^EX3Δ31/+1^ ) iPSC lines. HSP90 served as a loading control. (E) Bar graphs showing the relative levels of ^14^ C precipitated by TCA from protein extracts from day-20 hepatocytes derived from wild-type ( *MMUT* ^+/+^ ) and mutant ( *MMUT* ^EX3Δ17/+2^ , *MMUT* ^EX3Δ10/+1^ , and *MMUT* ^EX3Δ31/+1^ ) iPSCs (left panel). The mean total protein content of the TCA precipitates did not differ significantly across samples (right panel). Data are represented as mean ± SD, n = 3 - 6 biological replicates.

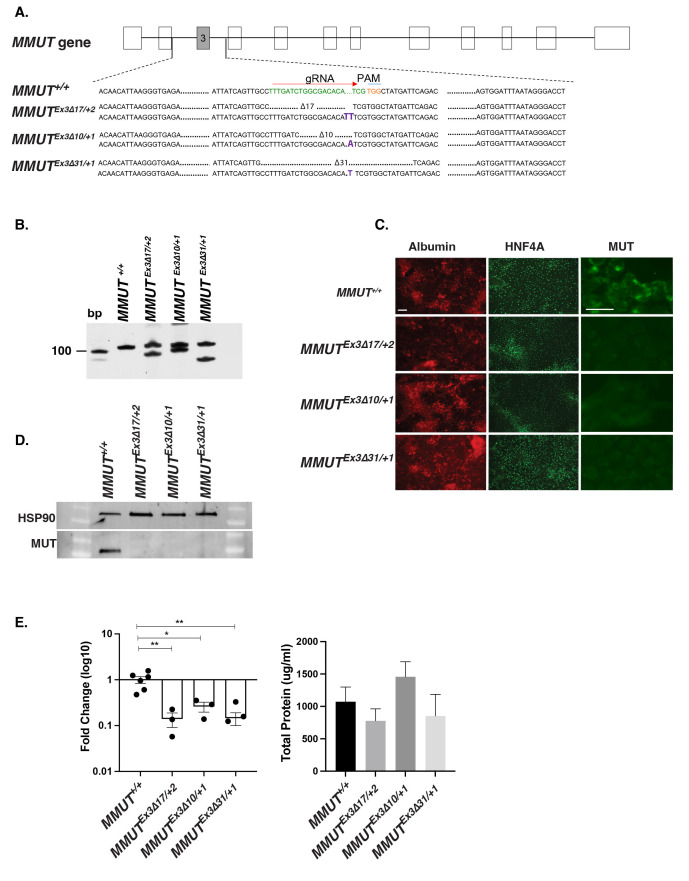



## Description

Methylmalonic acidemia (MMA) is an autosomal recessive disorder of the metabolism of branched-chain amino acids. It has been estimated that between 60-70% of MMA cases are caused by mutations in the *MMUT* gene that encodes the mitochondrial enzyme methylmalonyl-CoA mutase. (Habibzadeh et al., 2020). This enzyme catalyzes the conversion of methylmalonyl-CoA to succinyl-CoA (Fraser & Venditti, 2016). Affected patients fail to thrive and suffer from various clinical complications, including metabolic stroke, end-stage liver and renal failure, seizures, and developmental delays (Caterino et al., 2016) (Keyfi et al., 2016). Current treatments focus on limiting exposure to branched-chain amino acids through low-protein diets; however, these treatments fail to prevent substantial morbidity and mortality (Fraser & Venditti, 2016). Recent advances in mRNA (An et al., 2017) and gene therapy in animal models show some promise (Chandler & Venditti, 2019) (Senac et al., 2012); Unfortunately, for severely affected patients, liver transplants are the only beneficial option (Sen et al., 2023). The limitations of available therapeutics reflect a lack of models suitable for studying the molecular mechanisms of MMA and for identifying therapeutics (Hansen & Horslen, 2008). Although some mouse models of MMA can survive long-term (Chandler et al., 2022), differences between mice and humans limit their value. Human pluripotent stem cells that can be induced to differentiate into hepatocytes (Si-Tayeb, Noto, Nagaoka, et al., 2010) (Mallanna & Duncan, 2013) offer considerable potential as an alternative to rodents for modeling rare inborn errors in human metabolism (Musunuru, 2013) and as a platform for drug discovery.

We used CRISPR/Cas9 genome editing ( Figure 1A ) to generate a series of isogenic iPSCs harboring an *MMUT* gene mutation. A CRISPR guide RNA was designed to target exon 3 of *MMUT,* ensuring that any resulting frameshift mutations would inactivate enzyme activity, which is encoded by exons 8-12. Clonal cell lines were established and confirmed to express pluripotency markers via flow cytometry. PCR analyses ( Figure 1B ) and DNA sequencing ( Figure 1A ) were used to identify mutations. Three independent compound heterozygous lines were retained: *MMUT* ^Ex3∆17/+2^ , has a 17-base pair deletion in one allele and a 2-base pair insertion in the other; *MMUT* ^Ex3Δ10/+1^ , has a 10-base pair deletion and a 1-base pair insertion; and *MMUT* ^Ex3Δ31/+1^ , has a 31-base pair deletion and a 1-base pair insertion. All alleles were predicted to cause frameshifts leading to premature stop codons downstream of Exon 3: *MMUT* ^Ex3∆17/+2^ (p.Phe138SerfsTer141; Arg144PhefsTer180), *MMUT* ^Ex3Δ10/+1^ (p.Ala141ValfsTer176; His143GlnfsTer147), and *MMUT* ^Ex3Δ31/+1^ (p.Ala137ValfsTer167; Arg144SerfsTer147).

We compared the ability of MMUT-deficient and control *MMUT ^+/+^* iPSCs to differentiate into hepatocyte-like cells using our standard protocol, as previously described in detail (Si-Tayeb, Noto, Nagaoka, et al., 2010). After 20 days of differentiation, immunostaining showed that all cells strongly expressed characteristic hepatocyte markers, including Albumin and the transcription factor HNF4a ( Figure 1C ). Moreover, an anti-methylmalonase mutase antibody stained *MUT* ^+/+^ iPSC-derived hepatocytes, but no signal was detected in any of the mutant cells. We finally evaluated the effect of the mutations on MUT protein expression using immunoblot analysis. Figure 1D shows that the MUT protein was highly expressed in control cells but was undetectable in all three *MMUT* -disrupted lines ( Figure 1D ), consistent with predictions from DNA sequence analysis and the immunostaining experiments.

We next performed a ^14^ C-propionate fixation assay to determine whether mutations in MMUT restricted propionate metabolism. The ^14^ C-propionate fixation assay is a sensitive, reliable, and straightforward method for detecting inborn errors of propionate, methylmalonate, and cobalamin metabolism. This test is often employed to confirm MMA clinically, especially when genetic evidence is lacking (Forny et al., 2021). It works by providing ^14^ C-propionate to cells that rely on methylmalonyl-CoA mutase to facilitate the incorporation (fixation) of ^14^ C into acid-precipitable materials, including organic acids, amino acids, and proteins through the TCA cycle. Figure 1E shows that, although total protein levels were similar across all cells, ^14^ C fixation was markedly and significantly reduced in all three methylmalonyl-CoA mutase-deficient cells compared to the parental control *MMUT* ^+/+^ cells.

In summary, MMA is a serious disease caused by defects in the liver's ability to process branched-chain amino acids. Our data describe the creation of human iPSCs with mutations in the *MMUT* gene, which is the most common cause of MMA. We suggest that such cells could serve as a platform to better understand the role of hepatocytes in MMA and to help discover new treatments.

## Methods

**iPSC Lines and Culture**

Human K3 iPSCs were previously reprogrammed from human foreskin fibroblasts (Si-Tayeb, Noto, Sepac, et al., 2010). Pluripotent cells were cultured in low oxygen conditions (37°C, 4% O _2_ , 5% CO _2_ ) on an E-cadherin-IgG Fc fusion protein matrix (Nagaoka & Duncan, 2012), using defined mTeSR1 culture medium (Ludwig, Levenstein, et al., 2006) supplemented with zebrafish bFGF (40 ng/ml) (Ludwig, Bergendahl, et al., 2006). *MMUT* ^Ex3Δ17/+2^ , *MMUT* ^Ex3Δ10/+1^ , and *MMUT ^Ex3^* ^Δ31/+1^ cells were generated through CRISPR/Cas9 gene editing (Ran et al., 2013) (Heslop et al., 2021). Pluripotency was assessed by measuring OCT3/4 (Santa Cruz, USA) expression via flow cytometry using a Guava EasyCyte mini and differentiation to hepatocytes.

**CRISPR-mediated gene targeting**

CRISPR guide RNAs (gRNAs) targeting exon 3 of the *MMUT* gene were designed using an algorithm developed by Ran et al. Guide RNA sequences (Forward: CACCGTTTGATCTGGCGACACATCG; AAACCGATGTGTCGCCAGATCAAAC) were cloned into the PX459 pSPCas9(BB)-2A-Puro vector version 2 (Ran et al., 2013). K3 ( *MMUT* ^+/+^ ) iPSCs were seeded on to a Geltrex-coated (ThermoFisher/Gibco, CA, #A1413302) 10cm ^2^ tissue culture plate at 70-75% confluency. The following day, 60μg of the CRISPR/Cas9 plasmid containing gRNA was introduced into the pluripotent cells by electroporation. After 24 hours, 1 μg/ml of puromycin (Sigma Aldrich, MO, #P9620) was added to the culture medium for 48 hours to select for transfected cells. The surviving cell colonies were expanded, and samples from each were collected after 7-10 days for genomic DNA analyses. Indels were amplified using Herculase Fusion Polymerase (Agilent, CA, #600675), with the primer set (TGGTCAGCAGGGATTATCAG, CAGTGTCAATAGCATCTCCAGC). Amplicons were resolved by polyacrylamide gel electrophoresis using 4%–20% TBE gels (ThermoFisher/Invitrogen, CA, #EC6225). Potential INDELs were confirmed by DNA sequencing of amplicons and analyzed using TIDE (Brinkman & van Steensel, 2019).

**Differentiation of pluripotent stem cells**

 iPSCs were differentiated following a previously published protocol (Mallanna & Duncan, 2013). Briefly, cells were plated at 80% confluency on Matrigel-coated tissue culture plates. The next day, the media was replaced with RPMI medium containing B27 minus insulin (ThermoFisher Scientific, NY, #A1895601), Activin A (100 ng/ml; ThermoFisher Scientific, NY, #PHC9563), fibroblast growth factor 2 (FGF2, 20 ng/ml; ThermoFisher Scientific, NY, #PHG0023), and bone morphogenetic protein 4 (BMP4, 10 ng/ml; ThermoFisher Scientific, NY, #PHC9533) for 48 hours with daily media changes. To commit the populations to a definitive endoderm fate, cells were cultured in RPMI medium supplemented with B27 minus insulin and Activin A (100 ng/ml) for 48 hours, with daily media changes. Over the next five days, the cells were treated with B27 Supplement, 10 ng/mL FGF2, and 20 ng/mL BMP4 to convert them from definitive endoderm to hepatic progenitor cells. The cells were then cultured for an additional five days in B27 Supplement and 20 ng/mL Hepatocyte Growth Factor (Invitrogen, MA, #PHG0321) to generate immature hepatocytes. Finally, during the last five days of differentiation, they were induced to mature hepatocyte-like cells by culturing in HCM medium (Lonza, MD, #CC3198) supplemented with 20 ng/mL Oncostatin M (Invitrogen, MA, #PHC5015).

**14C propionate fixation assay**

The differentiated cells were treated with ^14^ C-propionate at a final concentration of 1 µCi/ml in hepatocyte medium overnight. The next day, both treated and untreated cells were enzymatically harvested with 0.25% trypsin-EDTA for 10 minutes at 37 °C after at two washes with PBS. The final pellet was resuspended in 5% TCA and incubated on ice for 30 minutes. The supernatant was removed by centrifugation, and 1N NaOH was added to make the samples soluble and ready for measurement of 14C incorporation using a Beckman Coulter 156500 scintillation counter (Willard et al., 1976).

**Immunofluorescence**

Cells were differentiated, washed once with PBS, and fixed with 4% paraformaldehyde (w/v; Santa Cruz, TX, #sc-281692) for 20 minutes. After washing the samples three times with PBS, they were permeabilized using 0.4% Triton X-100 for 20 minutes. Then, 3% BSA (w/v) in PBS was added for 1 hour to block the samples. The blocking solution was removed, and primary antibodies were applied and incubated overnight at 4°C in 1% BSA (w/v). The antibodies used included HNF4A (Santa Cruz, #sc-6556, 1:100) and Albumin (Cedarlane, #CL2513A, 1:500). The next day, the cells were washed three times with 1% BSA (w/v) in PBS and stained with DAPI (1 μg/ml; Sigma Aldrich, MO, #D1388) and AlexaFluor488 or AlexaFluor594 conjugated secondary antibodies (1:1000; ThermoFisher Scientific, NY) for 2 hours at room temperature. After three PBS washes, imaging was performed using a ZOE fluorescent cell imager (BioRad, CA). All images were processed identically. 

**Immunoblotting**

Cell lysates were collected using 1x RIPA buffer (Millipore, MA, #20-188) containing HALT protease inhibitor cocktail (ThermoFisher Scientific, NY, #78443) after washing the cells with PBS. Protein content was determined by a BCA assay (ThermoFisher/Pierce, IL, #23227). 10-30 μg of total protein was separated by SDS–PAGE in 4%–15% Mini-Protean TGX stain-free precast gels (BioRad, CA, #4568184). Separated proteins were transferred to PVDF membranes using the Trans-Blot Turbo Transfer System (BioRad, CA, #1704155). Membranes were blocked with 5% BSA (w/v) in TBS with 0.1% Tween (TBS-T) for one hour at room temperature. They were incubated overnight in 1% BSA in TBS-T at 4°C with antibodies against MUT (Santa Cruz, #sc-390978, 1:200) and HSP90b (Abcam, #ab32568, 1:100000). The next day, membranes were washed with 1% BSA (w/v) in TBS-T and then incubated with HRP-conjugated secondary antibodies for 2 hours at room temperature, followed by three washes with TBS-T. Clarity chemiluminescent reagent (BioRad, CA, #1705061) was applied for 1-5 minutes. Blot images were captured using the Chemidoc Touch gel imager (BioRad, CA). 

**Quantitative Real-Time PCR Analysis**

 RNA was isolated using the RNeasy Mini Kit following the manufacturer’s instructions (QIAGEN, #74106). RNA was treated with TURBO DNase (ThermoFisher/Ambion, NY, #AM1907) on column during RNA extraction. M-MLV Reverse Transcriptase (ThermoFisher/Invitrogen, NY, #28025-013) was used to generate cDNA. Quantitative real-time PCR (qRT-PCR) analysis was performed on a CFX384 real-time PCR machine (BioRad, CA) using TaqMan® Gene Expression assays (ThermoFisher/Applied Biosystems, NY, #4369016). Primer sets were purchased from Integrated DNA Technologies (Integrated DNA Technologies, IA).

**Quantification and statistical analysis**

Graphs were generated using Microsoft Excel or GraphPad Prism. Statistical analyses were performed using GraphPad Prism 8. For all analyses, experiments were performed in at least three independent biological replicates (n = independent biological replicates). Statistical significance between iPSC-derived cell lines was assessed using one-way ANOVA followed by Tukey’s post hoc multiple comparisons test.
